# A new approach to epitaxially grow high-quality GaN films on Si substrates: the combination of MBE and PLD

**DOI:** 10.1038/srep24448

**Published:** 2016-04-22

**Authors:** Wenliang Wang, Haiyan Wang, Weijia Yang, Yunnong Zhu, Guoqiang Li

**Affiliations:** 1 State Key Laboratory of Luminescent Materials and Devices, South China University of Technology, Guangzhou 510640, China; 2 Engineering Research Center on Solid-State Lighting and its Informationisation of Guangdong Province, South China University of Technology, Guangzhou 510640, China; 3 Department of Electronic Materials, School of Materials Science and Engineering, South China University of Technology, Guangzhou 510640, China

## Abstract

High-quality GaN epitaxial films have been grown on Si substrates with Al buffer layer by the combination of molecular beam epitaxy (MBE) and pulsed laser deposition (PLD) technologies. MBE is used to grow Al buffer layer at first, and then PLD is deployed to grow GaN epitaxial films on the Al buffer layer. The surface morphology, crystalline quality, and interfacial property of as-grown GaN epitaxial films on Si substrates are studied systematically. The as-grown ~300 nm-thick GaN epitaxial films grown at 850 °C with ~30 nm-thick Al buffer layer on Si substrates show high crystalline quality with the full-width at half-maximum (FWHM) for GaN(0002) and GaN(102) X-ray rocking curves of 0.45° and 0.61°, respectively; very flat GaN surface with the root-mean-square surface roughness of 2.5 nm; as well as the sharp and abrupt GaN/AlGaN/Al/Si hetero-interfaces. Furthermore, the corresponding growth mechanism of GaN epitaxial films grown on Si substrates with Al buffer layer by the combination of MBE and PLD is hence studied in depth. This work provides a novel and simple approach for the epitaxial growth of high-quality GaN epitaxial films on Si substrates.

Recently, due to their unique properties, such as direct band, high thermal stability, *etc*., GaN and its related III-nitrides have attracted considerable attention for the application in light-emitting diodes (LEDs), especially, liquid crystal display back-lighting, general lighting, and outdoor commercial display^1^,^2^,^3^.

By now, the GaN-based devices prepared on sapphire substrates have already been commercialized^4^,^5^. However, several obstacles originated from the sapphire substrates hamper the further development of GaN-based devices on sapphire substrates^6^,^7^. First, the sapphire has very low thermal conductivity, which means that it is poor in heat dissipation^6^. In this regard, the generated heat in the GaN-based devices prepared on sapphire substrates during working can’t be conducted out timely, and therefore leads to the poor performance and eventually short lifetime of GaN-based devices on sapphire substrates. Second, the size of commercially available sapphire is only of 4-inch, and it is costly^7^. Therefore, the price for the fabrication of GaN-based devices on sapphire substrates is very expensive.

To circumvent these issues, Si substrate seems to be a very promising material for the preparation of GaN-based devices. On the one hand, the thermal conductivity of Si is as high as 130 W/(m·K), which is 4 times higher than that of sapphire^8^,^9^. Therefore, heat dissipation of GaN-based devices grown on Si substrates will be very excellent. Second, the size of Si substrate is now available of 12-inch and its cost is very low, which benefits to the preparation of low cost GaN-based devices^8^,^9^. However, there are still several barriers in the growth of GaN epitaxial films on Si substrates. On the one hand, the direct growth of GaN on Si may lead to the formation of SiGa alloy or/and amorphous SiN during the high temperature growth by metal-organic vapor deposition (MOCVD), for example 1000 °C, which makes hard for the nucleation of GaN epitaxial films and thereby the failure of epitaxy^10^,^11^. On the other hand, the lattice and coefficient of thermal expansion (CTE) mismatches between GaN and Si are very large, which would lead to the formation of high-density dislocations formed during the initial growth and cooling process^12^,^13^. This is detrimental to the epitaxial growth of high-quality GaN epitaxial films. In this regard, a variety of approaches, such as AlN, SiC, and composition graded Al_x_Ga_1−x_N buffer layer, *etc.*, have been adopted to solve the meeting problems of GaN on Si mentioned above^10^,^14^,^15^,^16^. K.-L. Lin *et al.* used AlN as buffer layer to grow GaN epitaxial films on Si substrates^14^, and M. Wei *et al.* studied the effect of AlN buffer layer on the properties of GaN layer^16^; while D. Wang *et al.* deployed SiC as buffer layer to grow GaN epitaxial films on Si substrates^15^. In these two cases, due to the large lattice mismatch between buffer layer and Si substrate, high-quality GaN epitaxial films are hard to be obtained. Y. Lin *et al.* introduced composition graded Al_x_Ga_1−x_N buffer layer to grow GaN epitaxial films on Si substrates^10^. Although the high-quality GaN epitaxial films can be obtained, the process of growing composition grated Al_x_Ga_1−x_N buffer layer is very complicated, and the composition x in Al_x_Ga_1−x_N buffer layer is hard to be well controlled.

In this work, we report on a very effective and simple approach to grow GaN epitaxial films on Si substrates by the combination of MBE and PLD. MBE is deployed to grow ~30 nm-thick Al(111) epitaxial films on Si(111) substrates at first. On the one hand, due to the fact that the lattice mismatch between face centered cubic Al in [11–0] direction and diamond structure Si in [11–0] direction is as small as ~0.9%, the dislocations formed during the initial growth are greatly reduced when compared with that between AlN and Si, and eventually benefits to the nucleation of Al epitaxial films^17^,^18^. On the other hand, thanks to the advantageous of MBE, *i.e.* high-vacuum, high-quality Al(111) epitaxial films in single-crystalline can therefore be easily obtained on Si(111) substrates^17^,^18^, which is different from that grown by electron beam evaporation (EBE), where polycrystalline or amorphous Al epitaxial films are usually obtained owing to the inherent shortcoming of EBE system, *i.e.*, poor-vacuum^19^,^20^. After epitaxial growth of ~30 nm-thick Al buffer layer, PLD is then adopted to grow ~300 nm-thick GaN epitaxial films. Thanks to the kinetic energy of GaN precursors produced by PLD when ablating the GaN ceramic target, the generated GaN precursors would react with the Al atoms^21^,^22^, which leads to the formation of AlGaN layer to release the stress in the films. On the one hand, the lattice and CTE mismatches between AlGaN and GaN are very small^4^. On the other hand, during the growth of GaN epitaxial films, the kinetic energy GaN precursors produced by PLD have enough energy for the migration on the AlGaN buffer layer^21^,^22^. By the combination of MBE and PLD technologies, high-quality GaN epitaxial films on Si substrates with Al buffer layer have been obtained. The effect of growth temperature on the properties of as-grown GaN epitaxial films is studied, and the corresponding growth mechanism of GaN epitaxial films grown on Si substrates with Al buffer layer by the combination of MBE and PLD is hence proposed. This work provides an effective approach for the growth of high-quality GaN epitaxial films on Si substrates for the future application of GaN-based devices.

In order to obtain the oxide-free and hydrogen terminated Si surfaces in the air, the as-received Si substrates bought from Shanghai Daheng Optics and Fine mechanics Co., Ltd were cleaned by H_2_SO_4_ : H_2_O_2_ : H_2_O (3:1:1) and buffered-oxide-etch (BOE) HF (20:1), respectively. Before transferring into an ultra-high vacuum (UHV) growth chamber with a background pressure of 3.0 × 10^−10^ Torr, the as-cleaned Si(111) substrates were taken a degassing treatment in a UHV load-lock with a background pressure of 1.0 × 10^−8^ Torr for 30 min. Subsequently, the as-degassed Si(111) substrates were annealed at 850 °C for 60 min to remove the residual surface contaminations, which is beneficial to the subsequent growth of Al epitaxial films. During the epitaxial growth, ~30 nm-thick Al buffer layer was firstly grown on Si substrates at an optimized temperature of 750 °C by MBE with the Al cell temperature of 1200 °C. After the growth of Al buffer layer, ~300 nm-thick GaN epitaxial films were grown on Al buffer layer at the temperature ranging from at 550–850 °C by a KrF excimer laser light (λ = 248 nm, t = 20 ns) with optimized laser rastering program^21^,^22^, ablating the high-purity GaN (4N) target. Meanwhile, high-purity nitrogen with the optimized pressure of 4 mTorr was supplied through the inert gas purifier and the radio-frequency plasma radical generator was operated at 500 W to enhance the growth of GaN films. The laser energy was set at 250 mJ with a pulse repetition of 30 Hz. The as-grown GaN epitaxial films were studied by white-light interferometry (Y-Wafer GS4-GaN-R-405), *in-situ* reflection high energy electron diffraction (RHEED), scanning electron microscopy (SEM, Nova Nano SEM 430 Holland), atomic force microscopy (AFM, Bruker Dimension Edge, American), high-resolution X-ray diffraction (XRD, Bruker D8 X-ray diffractometer with Cu Kα1 X-ray source λ=1.5406 Å), and high-resolution transmission electron microscopy (TEM, JEOL 3000F, field emission gun TEM working at a voltage of 300 kV, which gives a point to point resolution of 0.17 nm) for surface morphology, crystalline quality, and interfacial property, respectively.

Figure 1a shows a typical photograph of ~300 nm-thick GaN film grown on Si(111) substrate at 850 °C for 90 min by PLD with optimized laser rastering program, and its corresponding thickness distribution measured by white-light interferometry is shown in Fig. 1b. From Fig. 1b, one can find that very homogeneous GaN epitaxial films with the thickness of ~300 nm are obtained. Therefore, the growth rate for GaN epitaxial films grown on Si substrates in this case is measured to be 200 nm/h. Using the same method, the growth rate for GaN epitaxial films grown on Si substrates at temperature of 550, 650, 750, 850 and 880 °C is determined to be 141, 168, 185, 200 and 208 nm/h, respectively, as shown in Fig. 1c. Evidently, with the increase in growth temperature from 550 to 880 °C, the growth rate for GaN epitaxial films on Si substrates is gradually increased.

The *in-situ* RHEED measurement is adopted to monitor the whole growth process. Figure 2a reveals a sharp and clear RHEED pattern for as-annealed Si(111) substrates, which confirms that atomically flat Si substrate surfaces are obtained. After annealing process, ~30 nm-thick Al epitaxial films are firstly grown on Si substrates by MBE. Figure 2b shows a spotted RHEED pattern for the Al epitaxial films, indicating that the Al epitaxial films with three-dimensional Al islands have been grown on Si substrates, which will be the nucleation centers for the subsequent films growth^22^. Finally, ~300 nm-thick GaN epitaxial films are grown on these Al epitaxial films. As for GaN epitaxial films grown at 550 °C, spotty RHEED patterns can be obtained, as shown in Fig. 2c. This is in striking contrast to that of GaN epitaxial films grown at 850 °C with identical thickness, Fig. 2d, revealing that the very flat GaN surface have been obtained. However, when the growth temperature for GaN epitaxial films is further increased to be 880 °C, the RHEED patterns for the ~300 nm-thick GaN epitaxial films become slightly poorer, as shown in Fig. 2e. After careful study, an in-plane epitaxial relationship of GaN[110]//Al[10]//Si[10] can be obtained^23^,^24^.

SEM and AFM measurements are deployed to study the surface morphology of as-grown GaN epitaxial films grown on Si substrates with ~30 nm-thick Al buffer layer at various temperatures. After annealing, the ~30 nm-thick Al epitaxial films are then grown on Si substrates. Figure 3a reveals that there are many islands formed on the Si surfaces with the diameter of 100–200 nm. Meanwhile, the AFM measurement confirms the results obtained from SEM measurement, revealing a RMS surface roughness of 16.1 nm shown in Fig. 4a. The height profile along a straight line on the as-grown Al surface indicates that the diameter of Al islands are in the range of 100–200 nm as illustrated in Fig. 4b, which is well consistent with SEM measurement. Subsequently, GaN epitaxial films are grown on Al buffer layer by PLD. Figure 3b shows the typical SEM image for the ~300 nm-thick GaN epitaxial films grown on Si substrates at 550 °C, where GaN islands homogenous distributed on Al layer with the diameter of 200–300 nm can be found, as shown in Fig. 3b. These results are proved by AFM measurement as illustrated in Fig. 4c,d. Obviously, the surface morphology for GaN epitaxial films grown on Si substrates at 550 °C is very rough, and its RMS surface roughness is measured to be 23.1 nm, as shown in Fig. 4c,d. As the growth temperature of GaN epitaxial films is increased, the surface morphology of as-grown GaN epitaxial films is improved significantly. Especially, GaN epitaxial films grown at 850 °C reveal a very smooth GaN surface, as shown in Fig. 3c, and the RMS surface roughness is reduced to 2.5 nm and the surface of GaN is coalesced, respectively, as illustrated in Fig. 4e,f. These are in striking contrast to the results obtained from the ~300 nm-thick GaN epitaxial films grown on Si substrates at 550 °C. However, when the growth temperature of GaN epitaxial films is further increased to 880 °C, the surface morphology for the as-grown GaN epitaxial films becomes slightly poorer with the RMS surface roughness of 3.8 nm, as shown in Fig. 3d, and Fig. 4g,h. Evidently, the growth temperature of GaN epitaxial films plays an important role in the surface morphology of GaN epitaxial films grown on Si substrates by PLD.

To further study the structural properties of as-grown GaN epitaxial films on Si substrates with the ~30 nm-thick Al buffer layer grown at various temperatures, XRD 2*θ*-*ω* and *φ* scans are adopted. Figure 5a reveals the 2*θ*-*ω* scan for the ~30 nm-thick Al epitaxial films grown on Si substrates, where only Al(111), Al(222), Si(111), Si(222) and Si(333) diffraction peaks can be found. This result confirms that the epitaxial relationship between Al and Si is Al(111)//Si(111). After growing Al epitaxial films, GaN epitaxial films are then grown on this layer. Figure 5b shows typical XRD 2*θ*-*ω* curve for the ~300 nm-thick GaN epitaxial films grown with temperatures ranging from 550 to 880 °C. From Fig. 5b, one can clearly identify that the intensity for GaN(0002) and GaN(0004) is gradually increased and then is slightly decreased as the growth temperature is raised from 550 to 880 °C, under the same Si signal as illustrated by red dashed frame in Fig. 5b. These results reveal the higher crystalline quality of the as-grown ~300 nm-thick GaN epitaxial films grown at 850 °C. To further study the structural properties of as-grown epitaxial films, a clearer XRD 2*θ*-*ω* curve for ~300 nm-thick GaN epitaxial films grown at 850 °C is provided in Fig. 5c, where the peaks of AlGaN(0002), AlGaN(0004) and Al(111) can be found. In this regard, the out-of-plane epitaxial relationship of GaN(0002)//AlGaN(0002)//Al(111)//Si(111) is obtained. In order to investigate the in-plane epitaxial relationship, typical *φ* scan is deployed. Due to the poor crystalline quality of Al and AlGaN epitaxial films, the *φ* scan for AlGaN and Al can’t be detected by XRD. In contrast to these results, six-fold rotational peaks for Si(11–3) and GaN(112–2) can be observed in Fig. 5d, which confirms that there are no 30° rotational domains existing between GaN epitaxial films and Si substrates. Meanwhile, an in-plane epitaxial relationship of GaN[112–0]//Si[11–0] can be obtained^25^,^26^. By the combination of XRD 2*θ*-*ω* and *φ* results, we therefore can conclude that GaN epitaxial films have been grown on Si substrates.

X-ray rocking curves (XRCs) are introduced to characterize the crystalline quality of the as-grown GaN epitaxial films. Figure 6a and b show the XRCs for GaN(0002) and GaN(101–2) of the ~300 nm-thick GaN epitaxial films grown at 850 °C, where the full-width at half-maximums (FWHMs) for GaN(0002) and GaN(101–2) XRCs of 0.45° and 0.61°, respectively, can be obtained. These results are in striking contrast to those of GaN(0002) and GaN(101–2) XRCs of 0.7° and 0.8°, respectively, obtained from the ~300 nm-thick GaN epitaxial films grown on Si substrates with AlN buffer layer by PLD and are also better than the GaN films grown on Si substrates with ZnO buffer layer by PLD^27^,^28^. It is known that the FWHMs of GaN(0002) and GaN(101–2) are related to the dislocation density, and the larger the FWHM is, the higher dislocation density in as-grown GaN epitaxial films will be^29^. Therefore, the crystalline quality of GaN epitaxial films grown on Si substrates with Al buffer layer is much better than that of GaN grown on Si substrates with AlN buffer layer by PLD.

The growth temperature dependence of crystalline quality for the ~300 nm-thick GaN epitaxial films grown with various temperatures is also studied. The FWHMs for GaN(0002) and GaN(101–2) XRCs are 1.2° and 1.75°, respectively, for the ~300 nm-thick GaN epitaxial films grown at 550 °C; which are gradually reduced to 0.45° and 0.61°, respectively, as the growth temperature increases to 850 °C, as shown in Fig. 6c. These results indicate that the crystalline quality increases as the growth temperature rises from 550 to 850 °C. However, as the growth temperature is further increased to 880 °C, the FWHMs for GaN(0002) and GaN(101–2) XRCs are 0.51° and 0.80°, indicating the incline in crystalline quality.

We attribute the smooth surface and high crystalline quality of GaN epitaxial films on Si substrates with Al buffer layer by the combination of MBE and PLD to two aspects. One is the utilization of MBE and PLD, and the other is the suitable growth temperature. In the former case, thanks to the inherent advantages of MBE, *i,e.*, high-vacuum, and small lattice mismatch between Al and Si, high-quality Al epitaxial films in single crystalline have been grown, which is beneficial to the subsequent growth of GaN epitaxial films. PLD is then employed to grow ~300 nm-thick GaN epitaxial films. Due to the superior advantages of PLD, the GaN precursor formed by PLD is highly-energetic, which has enough kinetic energy for the reaction of GaN to form AlGaN layer. The formation of AlGaN layer not only can release the formed stress in the films but also would benefit to the subsequent growth GaN epitaxial films due to the small lattice and CTE mismatches between GaN and AlGaN. In the latter case, suitable growth temperature is good to obtain suitable growth rate for the GaN growth, which helps the migration of GaN precursors to the equilibrium position, and eventually the high-quality GaN epitaxial films. If the growth temperature is too low, the growth rate for the GaN epitaxial films growth will be low. In this case, the GaN precursor is superimposed by the additional coming GaN precursor before moving into its equilibrium position due to the fact that the GaN precursor does not have enough kinetic energy^30^,^31^,^32^. If the growth temperature is too high, the growth rate for the GaN epitaxial films growth will be high. In this case, there is no sufficient time for the GaN precursor to migrate to its equilibrium position before being superimposed by the additional coming precursor. Both of these two cases would roughen the as-grown GaN surface 30,31,32. As a matter of fact, high-density dislocations are formed during these processes.

Cross-sectional TEM is adopted to study the interfacial property of the as-grown GaN epitaxial films grown on Si substrates with the ~30 nm-thick Al buffer layer. Figure 7a shows a cross-sectional TEM image of GaN epitaxial films grown on Si substrates at low magnification, where four layers, *i.e.,* Si substrates, buffer layer, GaN epitaxial films, and glue layer, from downside to upside can be clearly identified. Meanwhile, the thickness of GaN epitaxial films is measured to be ~301 nm, which is well consistent with the result obtained from white-light interferometry measurement. Figure 7b reveals a cross-sectional TEM image for GaN epitaxial films grown on Si substrates at medium magnification, from which ~30 nm-thick Al buffer layer can be found. To further study the Al/Si, AlGaN/Al and GaN/AlGaN hetero-interfaces, cross-sectional TEM image at high magnification is conducted, as shown in Fig. 7c–e, where sharp and abrupt Al/Si, AlGaN/Al and GaN/AlGaN hetero-interfaces can be clearly identified. Moreover, the formation of AlGaN layer is also proved, which is formed during the interfacial reactions between Al epitaxial films and GaN precursors during the initial growth. Selected area electron diffraction (SAED) is deployed to study these hetero-interfaces, as illustrated in Fig. 7f. From Fig. 7f, one can find the sharp and clear diffraction patterns for Si, Al, AlGaN and GaN, respectively, as marked in white, red, green, and blue circles, respectively. Meanwhile, some extra spots marked with pink circles are the diffractions from stacking faults of AlGaN^36^, ^37^,^38^,^39^,^40^. Additionally, after carefully study of SAED patterns, an in-plane epitaxial relationship of GaN[11–00]//AlGaN[11–00]//Al[112–]//Si[112–] can be obtained^21^,^22^,^33^,^34^,^35^,^36^.

Based on the characterizations mentioned above, the growth mechanism of GaN epitaxial films grown on Si substrates with Al buffer layer by the combination of MBE and PLD technologies is hence proposed. At first, Al buffer layer is grown on Si substrates by MBE, as shown in Fig. 7c. Due to the inherent advantages of MBE, *i.e.*, high-vacuum, and small lattice mismatch between Al and Si, high-quality Al epitaxial films can be grown on Si(111) substrates and is good for the subsequent growth of GaN epitaxial films, which is in striking contrast to the Al epitaxial films grown by EBE that usually shows poor-quality^19^,^20^. Afterwards, GaN epitaxial films are grown on these Al epitaxial films by PLD. It is known that the GaN precursor produced by PLD is usually highly-energetic and has enough energy for the reaction with Al buffer layer to form AlGaN, as shown in Fig. 7d. This process is in striking contrast to that of GaN grown by MOCVD or MBE, which does not form AlGaN when growing GaN on Al due to the fact that the GaN precursors produced by MOCVD and MBE are not highly-energetic enough^10^,^15^. One can find that there are stacking faults formed in AlGaN layer marked with red dashed frames in Fig. 7d,e. As for the nature of stacking fault formation, the intrinsic stacking fault character with a stacking sequence of …ABABCBCB… along the close-packed growth can be clearly identified from Fig. 7d, which implies that the stacking fault has introduced a thin layer of cubic stacking …ABC… into the hexagonal layer of the stacking sequence …ABABAB….^37^,^38^,^39^. In this regard, this stacking fault can be regarded as a thin plate like cubic inclusion in the hexagonal GaN caused by the presence of Al, which is confirmed by the XRD 2*θ*-*ω* measurement. During this process, the stress formed in AlGaN layer will be released by the formation of stacking faults^37^,^38^,^39^,^40^,^41^. Meanwhile, the formed AlGaN layer will be beneficial to the growth of GaN epitaxial films, because the lattice and CTE mismatches between GaN and AlGaN is very small. After the formation of AlGaN layer with relatively high crystalline quality, the GaN precursors produced by PLD can then be easily grown. Thanks to the inherent advantages of PLD, the GaN precursors produced by PLD have enough energy and sufficient time to migrate to the equilibrium position before being superimposed by the additional coming GaN precursor through optimizing the growth conditions, especially, growth temperature, to obtain the suitable growth rate, and eventually obtaining high-quality GaN epitaxial films with flat GaN surface and high crystalline quality, as well as the abrupt hetero-interfaces. The detailed processes are shown in Fig. 8. In this regard, by the combination of MBE and PLD technology to make full use of their advantages, GaN epitaxial films with relatively high crystalline quality can be grown on Si substrates within a very thin thickness of ~300 nm.

In summary, high-quality GaN epitaxial films have been grown on Si substrates with Al buffer layer by the combination of MBE and PLD technology. In this work, MBE is used to grow ~30 nm-thick Al epitaxial films at first, and then PLD is deployed to grow ~300 nm-thick GaN epitaxial films on this Al buffer layer. The effect of growth temperature on the properties of GaN epitaxial films grown on Si substrates with Al buffer layer is carefully studied. It is found that when the growth temperature is increased from 550 to 880 °C, the properties of the as-grown ~300 nm-thick GaN epitaxial films are gradually improved at first and then deteriorated, and the as-grown ~300 nm-thick GaN epitaxial films grown at 850 °C show the optimal values. The surface roughness of the as-grown GaN epitaxial films grown at 850 °C is measured to be 2.5 nm, and the FWHMs for GaN(0002) and GaN(101–2) XRCs are 0.45° and 0.61°, respectively. Moreover, the growth mechanism for the combination of MBE and PLD growth of high-quality GaN epitaxial films on Si substrates with Al buffer layer is hence proposed. Furthermore, this work provides an effective and simple approach for the growth of high-quality GaN epitaxial films on Si substrates. Based on this research, future work should be focused on the preparation of GaN-based devices with these high-quality GaN epitaxial films.

## Additional Information

**How to cite this article**: Wang, W. *et al.* A new approach to epitaxially grow high-quality GaN films on Si substrates: the combination of MBE and PLD. *Sci. Rep.* **6**, 24448; doi: 10.1038/srep24448 (2016).

## Figures and Tables

**Figure 1:**
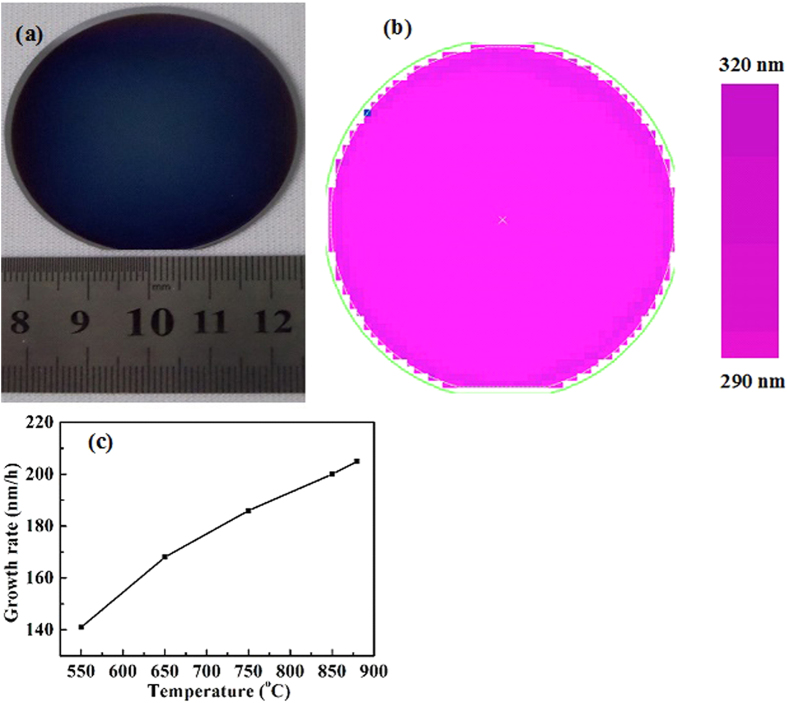
(**a**) Typical photograph of the ~300 nm-thick GaN epitaxial films grown on Si substrates at 850 °C, and (**b**) their thickness distribution measured by white-light interferometry. (**c**) The growth rate for the GaN epitaxial films grown on Si substrates with various temperatures ranging from 550 to 880 °C.

**Figure 2:**
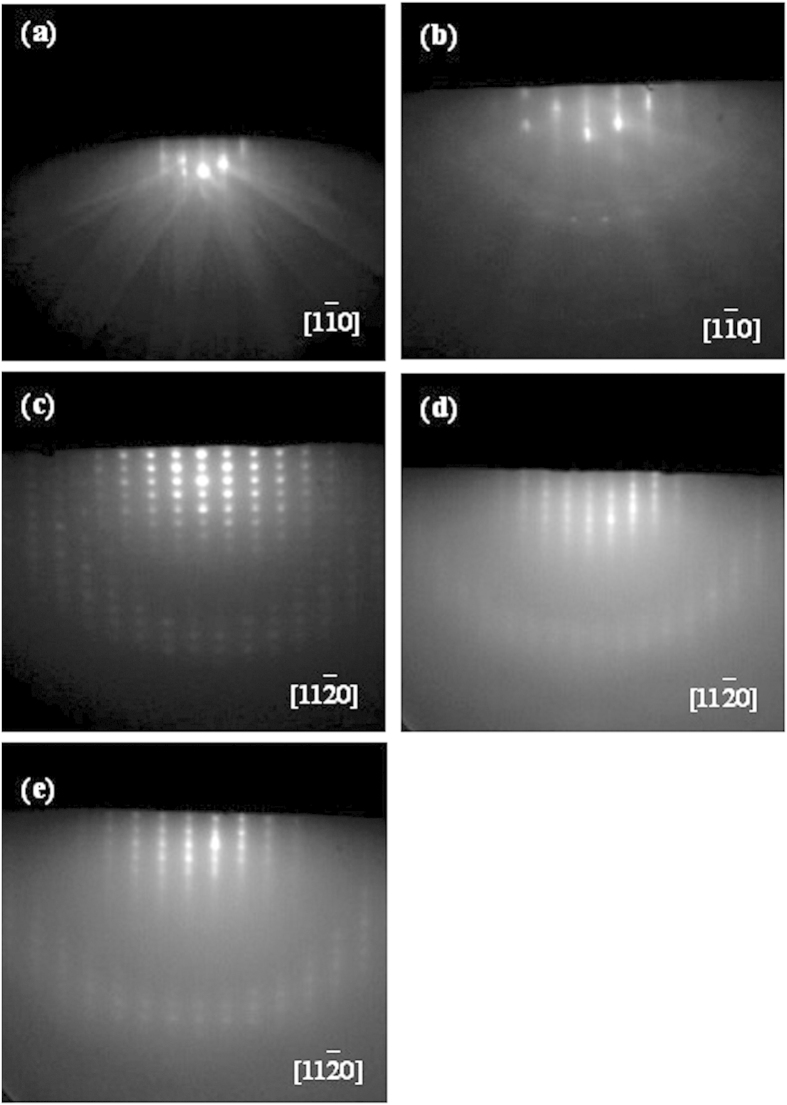
RHEED patterns for (**a**) the as-annealed Si substrates, (**b**) the ~30 nm-thick Al epitaxial films, and the ~300 nm-thick GaN epitaxial films grown at (**c**) 550, (**d**) 850 and (**e**) 880 °C, respectively.

**Figure 3:**
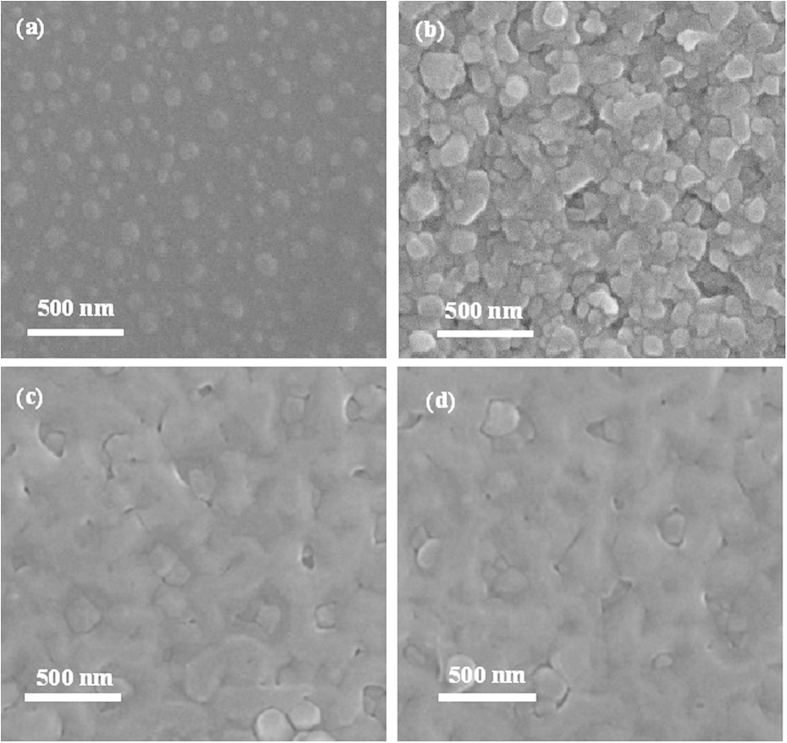
SEM images for (**a**) the as-annealed Si substrates, (**b**) the ~30 nm-thick Al epitaxial films, and the ~300 nm-thick GaN epitaxial films grown at (**c**) 550, (**d**) 850, and (**e**) 880 °C, respectively.

**Figure 4:**
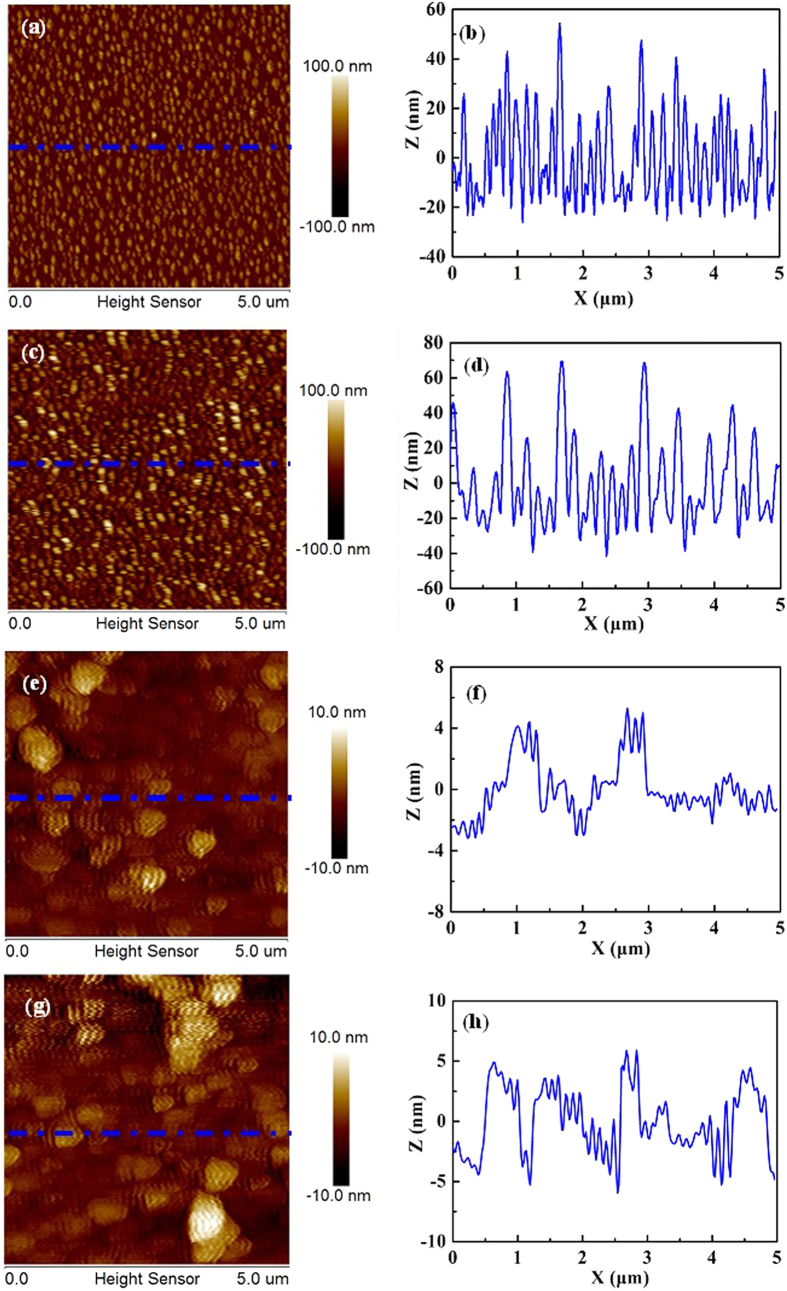
(**a**) AFM images for the ~30 nm-thick Al epitaxial films and (**b**) its corresponding height profile. (**c**) AFM images for the ~300 nm-thick GaN epitaxial films grown at 550 °C and (**d**) its corresponding height profile. (**e**) AFM images for the ~300 nm-thick GaN epitaxial films grown at 850 °C and (**f**) its corresponding height profile. (**g**) AFM images for the ~300 nm-thick GaN epitaxial films grown at 880 °C and (**h**) its corresponding height profile.

**Figure 5:**
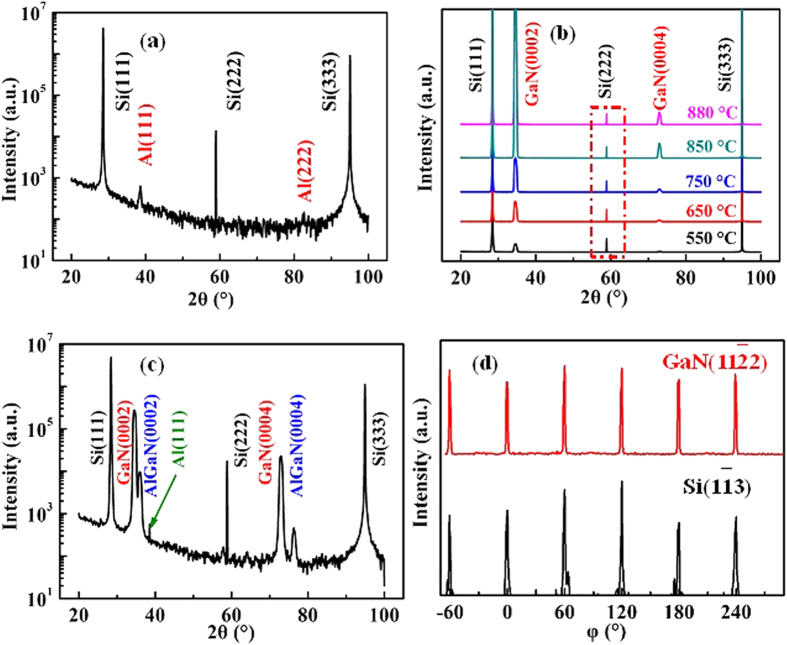
(**a**) Typical XRD 2*θ*-*ω* scans for the ~30 nm-thick Al buffer layer. (**b**) Temperature dependence of typical XRD 2*θ*-*ω* scans for the ~300 nm-thick GaN epitaxial films grown on Si substrates at various temperatures ranging from 550 to 880 °C and (**c**) the magnified XRD 2*θ*-*ω* scans for the ~300 nm-thick GaN epitaxial films grown on Si substrates at the temperature of 850 °C. (d) XRD *φ* scans for Si(13) and GaN(112), respectively.

**Figure 6:**
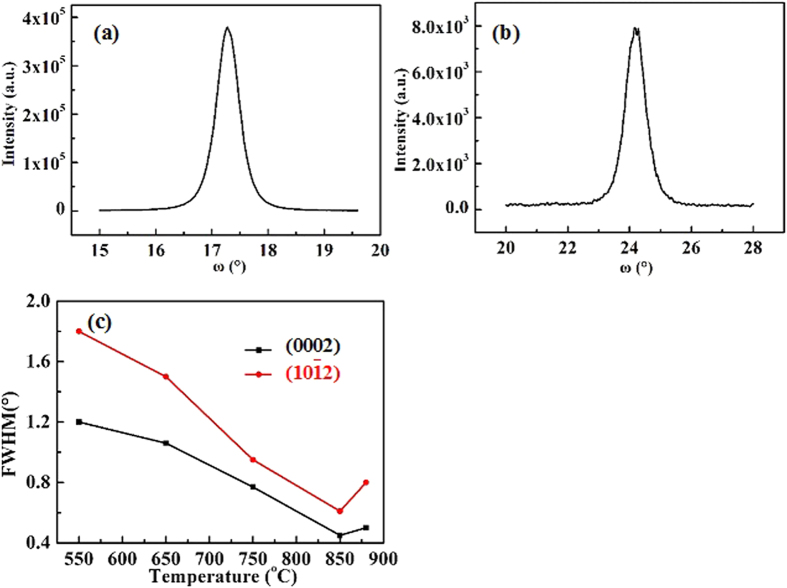
X-ray rocking curves of (**a**) GaN(0002) and (**b**) GaN(102) for the ~300 nm-thick GaN epitaxial films grown at 850 °C, respectively. (**c**) Temperature dependence of FWHMs of GaN(0002) and GaN(102) for the ~300 nm-thick GaN epitaxial films grown on Si substrates at various temperatures ranging from 550 to 850 °C.

**Figure 7:**
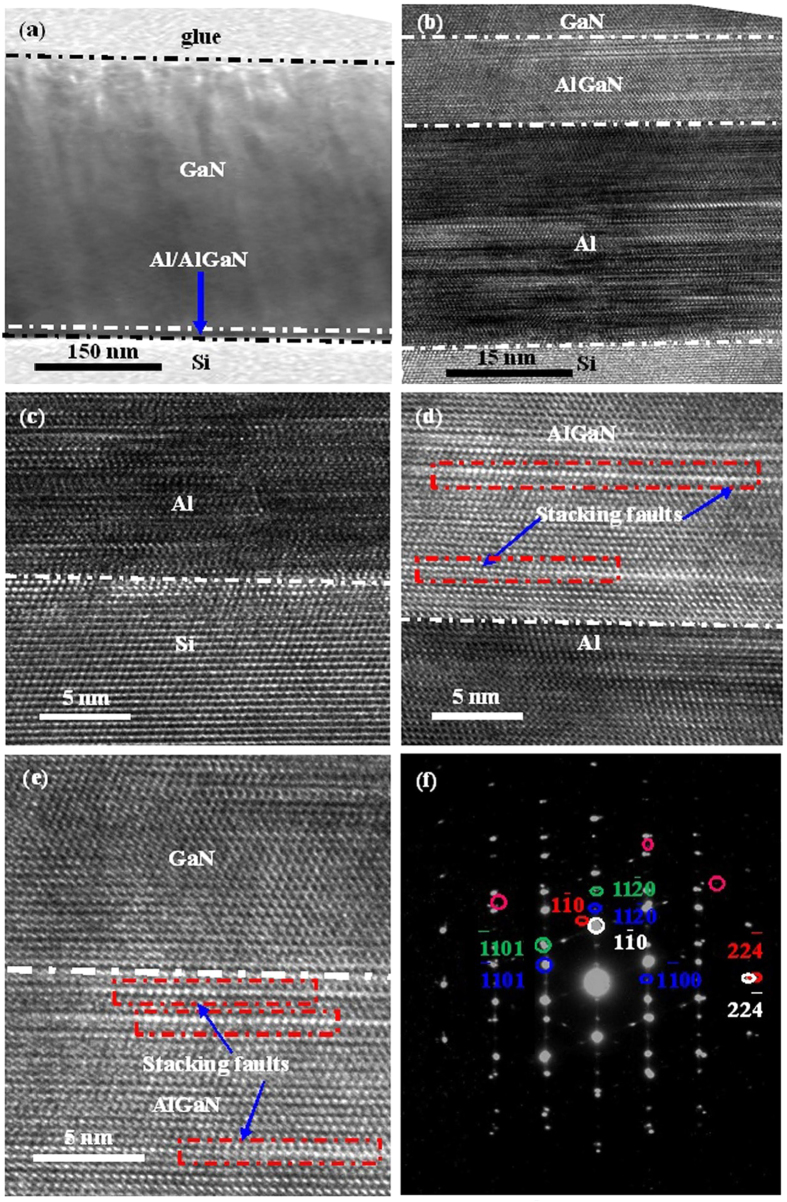
Cross-sectional high-resolution TEM images for the ~300 nm-thick GaN epitaxial films grown on Si substrates at 850 °C with a ~30 nm-thick Al buffer layer at (**a**) low magnification and (**b**) medium magnification. High-resolution TEM images for (**c**) the Al/Si hetero-interfaces, (**d**) the Al/AlGaN hetero-interfaces and (**e**) the AlGaN/GaN hetero-interfaces. (**f**) The SAED pattern for the ~300 nm-thick GaN epitaxial films grown on Si substrates with the ~30 nm-thick Al buffer layer, where the spots marked in white, red, green, blue and pink correspond to plane of Si, Al, AlGaN, GaN and stacking faults of AlGaN, respectively.

**Figure 8:**
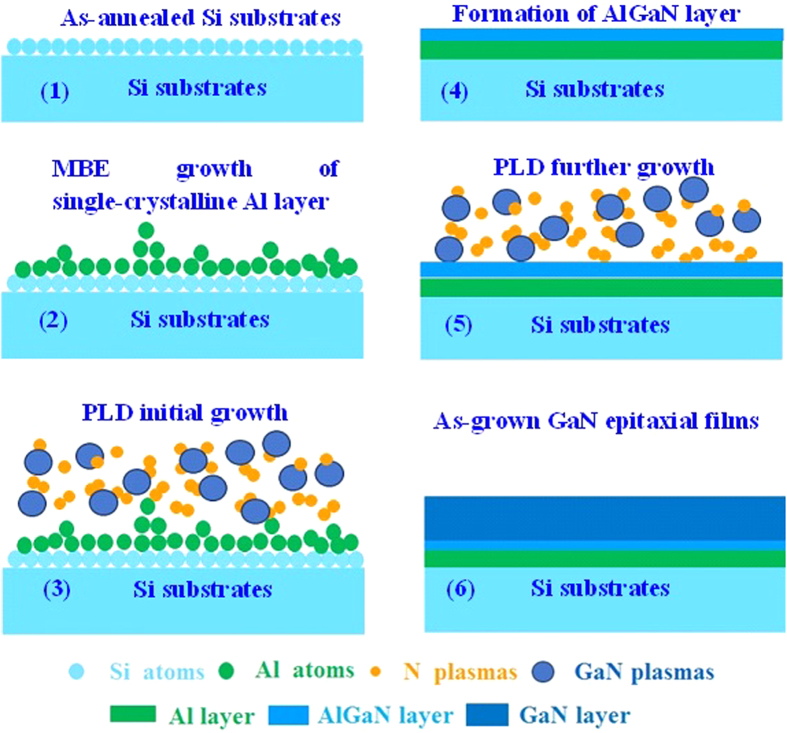
Schematic diagrams for the ~300 nm-thick GaN epitaxial films grown on Si substrates with the ~30 nm-thick Al buffer layer at 850 °C.

## References

[b1] Cheng C.-H. *et al.* Growing GaN LEDs on amorphous SiC buffer with variable C/Si compositions. Sci. Rep. 6, 19757 (2016). 26794268 10.1038/srep19757 PMC4726127

[b2] Camacho-Mojica D. C. & López-Urías F. GaN haeckelite single-layered nanostructures: monolayer and nanotubes. Sci. Rep. 5, 17902 (2015). 26658148 10.1038/srep17902 PMC4674713

[b3] Wei T. *et al.* Selectively grown photonic crystal structures for high efficiency InGaN emitting diodes using nanospherical-lens lithography. Appl. Phys. Lett. 101, 211111 (2012).

[b4] Wang W., Yang W., Wang H. & Li G. Epitaxial growth of GaN films on unconventional oxide substrates. J. Mater. Chem. C 2, 9342–9358 (2014).

[b5] Ra Y.-H., Navamathavan R., Park J.-H. & Lee C.-R. Radial growth behavior and characteristics of *m*-plane In_0.16_Ga_0.84_N/GaN MQW nanowires by MOCVD. CrystEngComm 15, 1874–1881 (2013).

[b6] Kukushkin S. A. *et al.* Substrates for epitaxy of gallium nitride: new materials and techniques. Rev. Adv. Mater. Sci. 17, 1–32 (2008).

[b7] Radtke G., Couillard M., Botton G. A., Zhu D. & Humphreys C. J. Structure and chemistry of the Si(111)/AlN interface. Appl. Phys. Lett. 100, 011910 (2012).

[b8] Zhu D., Wallis D. J. & Humphreys C. J. Prospects of III-nitride optoelectronics grown on Si. Rep. Prog. Phys. 76, 106501 (2013). 24088511 10.1088/0034-4885/76/10/106501

[b9] Bourret A., Barski A., Rouvière J. L., Renaud G. & Barbier A. Growth of aluminum nitride on (111) silicon: microstructure and interface structure. J. Appl. Phys. 83, 2003–2009 (1998).

[b10] Lin Y. *et al.* Performance improvement of GaN-based light-emitting diodes grown on Si(111) substrates by controlling the reactor pressure for the GaN nucleation layer growth. J. Mater. Chem. C 3, 1484–1490 (2015).

[b11] Liu R., Ponce F. A., Dadgar A. & Krost A. Atomic arrangement at the AlN/Si (111) interface. Appl. Phys. Lett. 83, 860–863 (2003).

[b12] Xiang R. F. *et al.* High quality GaN epilayers grown on Si (111) with thin nonlinearly composition-graded Al_x_Ga_1−x_N interlayers *via* metal-organic chemical vapor deposition. J. Alloy. Compd. 509, 2227–2231 (2011).

[b13] Kumar M. *et al.* Indium flux, growth temperature and RF power induced effects in InN layers grown on GaN/Si substrate by plasma-assisted MBE. J. Alloy. Compd. 513, 6–9 (2012).

[b14] Lin K.-L. *et al.* MOVPE high quality GaN film grown on Si (111) substrates using a multilayer AlN buffer. Phys. Stat. Sol. (c) 5, 1536–1538 (2008).

[b15] Wang D., Hiroyama Y., Tamura M., Ichikawa M. & Yoshida S. Growth of hexagonal GaN on Si(111) coated with a thin flat SiC buffer layer. Appl. Phys. Lett. 77, 1846–1848 (2000).

[b16] Wei M. *et al.* Effect of AlN buffer thickness on GaN epilayer grown on Si(111). Mater. Sci. Semicon. Proc. 14, 97–100 (2011).

[b17] Liu H. *et al.* Two-dimensional growth of Al films on Si(111)-7 × 7 at low-temperature. Surf. Sci. 571, 5–11 (2004).

[b18] Kotlyar V. G. *et al.* Formation of the ordered array of Al magic clusters on Si(111) −7 × 7. Phys. Rev. B 66, 165401 (2002).

[b19] Li Q. *et al.* Effect of flatness of Al film on fabrication of AAO template on silicon substrate. J. Wuhan Univ. 57, 38–42 (2011).

[b20] Huang J., Wang L., Shen Q., Lin C. & Östling M. Structural and electrical characterization of AlN thin films obtained by nitridation of Al/Si substrate. J. Electron. Mater. 28, 225–227 (1999).

[b21] Yang H., Wang W., Liu Z. & Li G. Epitaxial growth of 2 inch diameter homogeneous AlN single-crystalline films by pulsed laser deposition. J. Phys. D: Appl. Phys. 46, 105101 (2013).

[b22] Yang H., Wang W., Liu Z. & Li G. Homogeneous epitaxial growth of AlN single-crystalline films on 2 inch-diameter Si (111) substrates by pulsed laser deposition. Cryst Eng Comm 15, 7171–7176 (2013).

[b23] Ichimiya A. & Cohen P. I. Reflection high-energy electron diffraction (Cambridge University, 2004).

[b24] Wang W. *et al.* Synthesis of homogeneous and high-quality GaN films on Cu(111) substrates by pulsed laser deposition. CrystEngComm, 16, 8500–8507 (2014).

[b25] Moram M. A. & Vickers M. E. X-ray diffraction of III-nitrides. Rep. Prog. Phys. 72, 036502 (2009).

[b26] Shao Y. *et al.* EBSD crystallographic orientation research on strain distribution in hydride vapor phase epitaxy GaN grown on patterned substrate. Cryst Eng Comm 15, 7965–7969 (2013).

[b27] Wang W., Yang W., Lin Y., Zhou S. & Li G. Microstructures and growth mechanisms of GaN films epitaxially grown on AlN hetero-structures by pulsed laser deposition at different temperatures. Sci, Rep. 5, 16453 (2015). 26563573 10.1038/srep16453 PMC4643238

[b28] Hong J.-I., Chang Y., Ding Y., Wang Z. L. & Snyder R. L. Growth of GaN films with controlled out-of-plane texture on Si wafers. Thin solid films 519, 3608–3611 (2011).

[b29] Wang W., Yang H. & Li G. Growth and characterization of GaN-based LED wafers on La_0.3_Sr_1.7_AlTaO_6_ substrates. J. Mater. Chem. C 1, 4070–4077 (2013).

[b30] Capellini G., De Seta M. & Evangelisti F. Ge/Si(100) islands: Growth dynamics versus growth rate. J. Appl. Phys. 93, 291–295 (2003).

[b31] Krzyzewski T. & Jones T. Effects of growth rate on crystal perfection and lifetime in germanium. J. Appl. Phys. 96, 668–673 (2004).

[b32] Wang W. *et al.* Effect of Al evaporation temperature on the properties of Al films grown on sapphire substrates by molecular beam epitaxy. RSC Adv. 5, 29153–29158 (2015).

[b33] Ra Y.-H., Navamathavan R. & Lee C.-R. Growth characteristics of uniaxial InGaN/GaN MQW/n-GaN nanowires on Si(111) using MOCVD. Cryst Eng Comm 14, 8208–8214 (2012).

[b34] Wang W. *et al.* Epitaxial growth of high quality AlN films on metallic aluminum substrates. Cryst Eng Comm 16, 4100–4107 (2014).

[b35] Heying B. *et al.* Control of GaN surface morphologies using plasma-assisted molecular beam epitaxy. J. Appl. Phys. 88, 1855–1860 (2000).

[b36] Li G., Wang W., Yang W. & Wang H. Epitaxial growth of group III-nitride films by pulsed laser deposition and their use in the development of LED devices. Surf. Sci. Rep. 70, 380–423 (2015).

[b37] Kim H., Andersson T. G., Chauveau J.-M. & Trampert A. As-mediated stacking fault in wurtzite GaN epilayers. Appl. Phys. Lett. 81, 3407–3409 (2002).

[b38] Cho Y. S. *et al.* Reduction of stacking fault density in *m*-plane GaN grown on SiC. Appl. Phys. Lett. 93, 111904 (2008).

[b39] Vermaut P., Nouet G. & Ruterana P. Observation of two atomic configurations for the {110} stacking fault in wurtzite (Ga, Al) nitrides. Appl. Phys. Lett. 74, 694–696 (1999).

[b40] Liao X. Z. *et al.* Strain relaxation by alloying effects in Ge islands grown on Si(001). Phys. Rev. B 60, 15605 (1999).

[b41] Liao X. Z. *et al.* Alloying, elemental enrichment, and interdiffusion during the growth of Ge(Si)/Si(001) quantum dots. Phys. Rev. B 65, 153306 (2002).

